# 

**Published:** 1863-09

**Authors:** 


					﻿THE
DENTAL REGISTER
OF THE WEST.
EDITED BY
J. TAFT AND GEO. WATT.
SEPTEMIBER.-1863.
MONTHLY:
Three Dollars per Annum, in advance.
CINCINNATI :
J. TAFT, PUBLISHER.
T. Taft. Dentist. 172 I’ace Street.
				

